# Accuracy of ultrasonography in the diagnosis of acute calculous cholecystitis: review of the literature

**DOI:** 10.1186/2036-7902-5-S1-S11

**Published:** 2013-07-15

**Authors:** Antonio Pinto, Alfonso Reginelli, Lucio Cagini, Francesco Coppolino, Antonio Amato Stabile Ianora, Renata Bracale, Melchiore Giganti, Luigia Romano

**Affiliations:** 1 Department of Diagnostic Imaging, A. Cardarelli Hospital, Naples, Italy; 2 Department of Internal and Experimental Medicine, Magrassi-Lanzara, Institute of Radiology, Second University of Naples, Naples, Italy; 3 University of Perugia, Thoracic Surgery Unit, Perugia, Italy; 4 University of Palermo, Department of Radiology, Palermo, Italy; 5 University of Bari, Diagnostic Imaging Section, Bari, Italy; 6 Department of Health Science, University of Molise, Campobasso, Italy; 7 University of Ferrara, Dipartimento di Scienze Chirurgiche, Ferrara, Italy

**Keywords:** Acute calculous cholecystitis, Ultrasonography, Computed Tomography, Magnetic Resonance Cholangiopancreatography, Cholescintigraphy

## Abstract

**Background:**

To evaluate the accuracy of ultrasonography in the diagnosis of acute calculous cholecystitis in comparison with other imaging modalities.

**Methods:**

The authors performed a search of the Medline/ PubMed (National Library of Medicine, Bethesda, Maryland) for original research and review publications examining the accuracy of ultrasonography in the diagnosis of acute calculous cholecystitis. The search design utilized a single or combination of the following terms : (1) acute cholecystitis, (2) ultrasonography, (3) computed tomography, (4) magnetic resonance cholangiopancreatography and (5) cholescintigraphy. This review was restricted to human studies and to English-language literature. Four authors reviewed all the titles and subsequent the abstract of 198 articles that appeared appropriate. Other articles were recognized by reviewing the reference lists of significant papers. Finally, the full text of 31 papers was reviewed.

**Results:**

Sonography is still used as the initial imaging technique for evaluating patients with suspected acute calculous cholecystitis because of its high sensitivity at the detection of GB stones, its real-time character, and its speed and portability. Cholescintigraphy still has the highest sensitivity and specificity in patients who are suspected of having acute cholecystitis. However, due to a combination of reasons including logistic drawbacks, broad imaging capability and clinician referral pattern the use of cholescintigraphy is limited in clinical practice. CT is particularly useful for evaluating the many complications of acute calculous cholecystitis. The lack of widespread availability of MRI and the relatively high cost prohibits its primary use in patients with acute calculous cholecystitis.

**Conclusions:**

US is currently considered the preferred initial imaging technique for patients who are clinically suspected of having acute calculous cholecystitis.

## Background

Acute cholecystitis is defined as an acute inflammation of the gallbladder wall, regardless of the cause. In the many of cases, the underlying etiology is the obstruction of the cystic duct due to an impacted stone in either the neck of the gallbladder or the cystic duct (acute calculous cholecystitis). Acute cholecystitis can also develop without associated cholelithiasis (acute acalculous cholecystitis).

It is very uncommon that a patient without an history of biliary symptoms, such as colic pain, develop an acute cholecystitis. Conversely, < 15% of the patients with cholelithiasis experience clinical symptoms and < 5% an acute cholecystitis [1].

An estimated 25 million Americans have cholelithiasis. In 80% of cases, the cholelithiasis is primarily composed of cholesterol, with pigments, calcium bilirubinate, and calcium carbonate accounting for most of the remainder [2,3].

Forty percent of patients with acute cholecystitis develop complications [4]: the complications of cholecystitis most commonly result from the impaction of gallstones in the cystic duct or common bile duct and include gallbladder hydrops, emphysematous cholecystitis, which is seen more commonly in men and diabetic patients, gangrenous cholecystitis and perforation of the gallbladder. The gallbladder may perforate into the abdominal cavity, causing peritonitis, or the perforation may be contained by the omentum leading to an intra-abdominal abscess.

Emergency conditions involving the gallbladder and the bile ducts are common radiological challenging problems. Imaging provides valuable informations for the following reasons: (1) to ensure the final diagnosis, as up to 20% of patients clinically classified as having acute cholecystitis have another disease that does not require surgery; (2) to prevent the patient from complications in case of delayed diagnosis and (3) to detect complications which may urge the surgical treatment [1].

In this paper we evaluate the accuracy of ultrasonography in the diagnosis of acute calculous cholecystitis in comparison with other imaging modalities through a literature search.

## Methods

The authors performed a search of the Medline/ PubMed (National Library of Medicine, Bethesda, Maryland) for original research and review publications examining the accuracy of ultrasonography in the diagnosis of acute calculous cholecystitis in comparison with other imaging modalities. The search design utilized a single or combination of the following terms : (1) acute cholecystitis, (2) ultrasonography, (3) cholescintigraphy, (4) computed tomography and (5) magnetic resonance cholangiopancreatography. This review was restricted to human studies and to English-language literature. Four authors reviewed all the titles and subsequent the abstract of 198 articles that appeared appropriate. Other articles were recognized by reviewing the reference lists of significant papers. Finally, the full text of 31 papers was reviewed.

## Results

### Acute calculous cholecystitis: diagnosis with conventional radiography

Stonelike densities may be single or multiple. They may be irregular, show a marginal location or may exhibit dense, uniform calcification.

Plain abdominal film is of limited value in the setting of gallbladder disease because only 15–20% of gallstones are visible on a radiograph of the abdomen and little information about complicated gallbladder disease can be obtained using conventional radiography [2].

### Acute calculous cholecystitis: diagnosis with ultrasonography

Ultrasound (US) is the preferred imaging examination for the diagnosis of acute cholecystitis and is the first method used when the clinical presentation is suggestive of biliary pathology. The main findings of acute calculous cholecystitis on US include in addition to the presence of stones: distension of the gallbladder lumen, gallbladder wall thickening, a positive US Murphy sign, pericholecystic fluid [5,6] and a hyperemic wall upon evaluation with Color Doppler [7,8].

Ultrasound has the best sensitivity and specificity for evaluating patients with suspected gallstones [9]. As reported in the literature [10], some ultrasonographic findings are more strongly associated with acute cholecystitis than others: a positive Murphy’s sign (pain is provoked by either the transducer or the sonographer’s palpation under guidance, in the exact area of the gallbladder) is reported to have sensitivity as high as 88% [11,12]. Ralls at al. [13]report that one of the most important advantages of ultrasound over other imaging techniques in the investigation of acute cholecystitis is the ability to assess for a sonographic Murphy sign, which is a reliable indicator of acute cholecystitis with a sensitivity of 92% [13]. An increased gallbladder wall thickness of > 3.5 mm has been found to be a reliable and independent predictor of acute cholecystitis [14].

Visualization of gallbladder wall thickening in the presence of gallstones using ultrasound has a positive predictive value of 95% for the diagnosis of acute cholecystitis [13].

Unfortunately, thickening of the gallbladder wall in the absence of cholecystitis may be observed in systemic conditions, such as liver, renal, and heart failure, possibly due to elevated portal and systemic venous pressures [15].

Moreover, as most patients are examined in the emergency setting, it has sometimes been questioned if the younger radiologists would perform as well as experienced radiologists, who are not always available at the time of examination. Grantcharov et al. [16] prospectively evaluated the interobserver agreement in US examination of the gallbladder and the biliary tract performed by an experienced and a novice radiologist: they report that the novice radiologist's expertise in the primary diagnosis of uncomplicated gallstone disease was as good as the one provided by the experienced colleague and the significant interobserver difference in the measurements of the thickness of the gallbladder wall and the common bile duct diameter might indicate that assessment of these parameters requires extensive practise.

### Acute calculous cholecystitis: diagnosis with cholescintigraphy

Cholescintigraphy is a method widely used in some countries and completely ignored in others. The principle is to show that the radiopharmaceuticals (diisopropyl-iminodiacetic acid, or DISIDA) fills in the gallbladder within 30 minutes in normal subjects. In case of cystic duct obstruction, gallbladder will not enhance, even after hours.

In 1994, Shea et al [9] reported a systematic review of imaging studies published between 1978 and 1990. In this review, they concluded that cholescintigraphy had the best sensitivity (97%; 95% confidence interval [CI]: 96%, 98%) and specificity (90%; 95% CI: 86%, 95%) in the detection of acute cholecystitis, whereas US had a sensitivity of 88% (95% CI: 74%, 100%) and a specificity of 80% (95% CI: 62%, 98%). Recently Kiewiet et al [17] update the diagnostic accuracy summary estimates for imaging modalities described by Shea et al [9], by using state-of-the-art methods for the meta-analysis of diagnostic accuracy studies: in this systematic review, they observed that cholescintigraphy has the highest diagnostic accuracy of all imaging modalities in the detection of acute cholecystitis.

### Acute calculous cholecystitis: diagnosis with Computed Tomography

As previoulsy reported, gallbladder sonography or cholescintigraphy is usually the first imaging study performed, because these modalities have been shown to be both sensitive and specific for this condition. However, some patients with acute cholecystitis have a confusing or complicated presentation and may be a diagnostic dilemma. In these patients, possible diagnoses include abscess, pancreatitis, ischemic bowel, or other abdominal inflammatory conditions. Many of these patients may be referred for a CT of the abdomen[18,19]. CT is particularly useful for evaluating the many complications of acute cholecystitis, such as emphysematous cholecystitis, gangrenous cholecystitis, hemorrhage, and gallstone ileus [20,21]. Moreover CT is also useful in making the specific diagnosis when obesity or gaseous distention limit the use of US.

### Acute calculous cholecystitis: diagnosis with Magnetic Resonance Imaging

Both conventional magnetic resonance imaging (MRI) [22-24] and magnetic resonance cholangiopancreatography [23,24] have been evaluated in the diagnosis of acute calculous cholecystitis and its complications [25]. When compared to ultrasonography, some have found MRI to be equivalent [26] and some have found it to be superior [27]. Park et al. report that in the assessment of acute cholecystitis, US is superior to MR cholangiography in the evaluation of gallbladder wall thickening. However, MR cholangiography is superior to US in the depiction of cystic duct and gallbladder neck calculi at the evaluation of cystic duct obstruction [28]. The lack of widespread availability of MRI and the relatively high cost prohibits its primary use for now [29-31].

## Discussion

Acute cholecystitis accounts for 3–10% of all patients with abdominal pain and is the most common cause of acute abdominal pain in the right upper quadrant, especially in the elderly patients [25]. Sonography is still used as the initial imaging technique for evaluating patients with suspected gallbladder (GB) disease because of its high sensitivity at the detection of GB stones, its real-time character, and its speed and portability [26].

Cholescintigraphy still has the highest sensitivity and specificity (96% and 90%) in patients who are suspected of having acute cholecystitis. However, due to a combination of reasons including logistic drawbacks, broad imaging capability and clinician referral pattern (especially in the emergency setting) the use of cholescintigraphy is limited in clinical practice [9] and US has emerged as the first-line imaging modality for the diagnosis of acute calculous cholecystitis. There is a lack of availability of equipment and/or personnel and the examination time takes up to several hours, whereas a full abdominal US examination is readily available, can be performed in 10–15 minutes, and allows for assessment of pain localized to the gallbladder region (sonographic Murphy sign). Furthermore, cholescintigraphy provides information confined to the hepatobilliary tract, whereas US can be useful in diagnosing other pathologic conditions responsible for the abdominal complaints. Cholescintigraphy also carries the burden of ionizing radiation whereas US and MR imaging do not [15].

## Limitations

There are several limitations to our study. Some relevant to this review include: accuracy, unobtainable texts and timeframe restrictions. In this review we do not used statistical techniques for combining results of the eligible studies. Another important limitation is the limited number of recent studies in which a head-to-head comparison has been performed.

## Conclusions

US is currently considered the preferred initial imaging technique for patients who are clinically suspected of having acute calculous cholecystitis. US is preferred by the majority of radiologists because of lower costs, better availability after hours, and more evidence regarding its accuracy for cholecystitis. In current practice, in the emergency setting, CT is being used increasingly especially in elderly patients with abdominal pain even when they are suspected of having acute cholecystitis.

## Competing interests

The authors declare that they have no competing interests.

## Authors’ contributions

AP participated in the conception, design, and coordination of the study as well as in the acquisition and analysis of the data and helped draft the manuscript. AR participated in the acquisition and analysis of the data as well as in the drafting and revision of the manuscript for submission. LC, FC, AASI, B, MG participated in the conception and design of the study and in the revision of the manuscript. MZ participated in the conception and design of the study and in the revision of the manuscript. LR participated in the acquisition and analysis of the data as well as in the drafting and revision of the manuscript for submission.

All authors read and approved the final manuscript.

## Declarations

This article has been published as part of *Critical Ultrasound Journal* Volume 5 Supplement 1, 2013: Topics in emergency abdominal ultrasonography. The full contents of the supplement are available online at http://www.criticalultrasoundjournal.com/supplements/5/S1. Publication of this supplement has been funded by the University of Molise, University of Siena, University of Cagliari, University of Ferrara and University of Turin.
